# Noninvasive Predictors of High-Risk Varices in Patients with Non-Cirrhotic Portal Hypertension

**DOI:** 10.1155/2019/1808797

**Published:** 2019-02-07

**Authors:** Morven E. Cunningham, Gilda Parastandeh-Chehr, Orlando Cerocchi, David K. Wong, Keyur Patel

**Affiliations:** Toronto Centre for Liver Disease, University Hospital Network, Toronto M5G 2C4, Canada

## Abstract

Non-cirrhotic portal hypertension (NCPH) comprises a heterogeneous group of liver disorders causing portal hypertension without cirrhosis and carries a high risk of variceal bleeding. Recent guidelines, based largely on patients with viral cirrhosis, suggest low likelihood of high risk varices (HRV) in patients with a liver stiffness measurement (LSM) <20 kPa and platelet count >150 × 10^9^/L. In NCPH, LSM is often higher than healthy controls but lower than matched cirrhotic patients. The aim of this study was to assess whether LSM or other noninvasive assessments of portal hypertension could predict HRV in NCPH patients.* Methods*. Records of patients with NCPH seen at a single centre between 2007 and 2018 were reviewed retrospectively. Primary outcome measure was presence or absence of HRV at gastroscopy within 12 months of clinical assessment. Association of LSM or other clinical features of portal hypertension (spleen size, platelet count, platelet count/spleen length ratio (PSL), LSM-spleen length/platelet count ratio score (LSP)) with HRV and ability of these variables to predict HRV was analysed.* Results*. Of 44 patients with NCPH who met inclusion criteria, 34% (15/44) had HRV. In a multivariate model, spleen size and PSL correlated with HRV but platelet count, LSM, and LSP did not (spleen size: *β* = 0.35,* p *= 0.02; OR 1.42, 95% CI 1.06-1.92; PSL: *β* = -1.47,* p *= 0.02; OR 0.23, 95% CI 0.07-0.80). There was no significant difference between spleen size and PSL in predicting HRV (AUROC 0.81 (95% CI 0.66 – 0.91) versus 0.71 (95% CI 0.54 – 0.84), respectively,* p* = 0.400). Spleen size >17.2cm had sensitivity 78.6% and specificity 64.3% for prediction of HRV.* Conclusions*. In NCPH patients, spleen size may predict risk of HRV at gastroscopy within 12 months. LSM and platelet count are not useful to assess risk of HRV in NCPH.

## 1. Introduction

Non-cirrhotic portal hypertension (NCPH) comprises a heterogeneous group of liver disorders which result in portal hypertension in the absence of cirrhosis and carries a high risk of variceal bleeding. In Western countries, portal hypertension is typically the result of cirrhosis, with NCPH accounting for less than 10% of cases. In other parts of the world, NCPH is the leading cause of portal hypertension [1]. The commonest causes of NCPH are schistosomiasis and portal vein thrombosis, but a diverse range of other causes are recognised, including infiltrative liver diseases, autoimmune disease, immunodeficiency syndromes, drug reactions, and congenital liver diseases, as well as idiopathic NCPH. Portal hypertension is often asymptomatic until complications develop. Among asymptomatic patients with cirrhosis, 80 to 90% will have an elevated portal pressure, 40% of whom will have oesophageal varices. Among those who do not have varices, these may develop at a rate of 6-10% per year, depending on the severity of portal hypertension [1].

Over recent years, liver stiffness measurement (LSM) by transient elastography has become increasingly recognised as a noninvasive tool to establish the presence of cirrhosis in liver disease of varying aetiologies [2]. The development of portal hypertension is an important complication of cirrhosis as it is associated with increased risk of complications of liver disease and poorer prognosis. The gold standard for diagnosis of portal hypertension remains measurement of hepatic venous pressure gradient (HVPG). HVPG >10mmHg defines clinically significant portal hypertension in cirrhosis, as pressures at this level and above are independently associated with both hepatic decompensation and development of HCC [3, 4]. In patients with cirrhosis, LSM correlates with portal hypertension up to HVPG of 12 mmHg, after which the correlation is lost, likely due to the influence of extrahepatic factors which perpetuate splanchnic vasodilatation and portal hypertension, leading to altered hepatic blood flow and sinusoidal congestion, independent of the degree of hepatic fibrosis [5].

The role of LSM in NCPH is less clear. There is very little data on whether LSM correlates with portal hypertension in NCPH, although in one small series of patients with nodular regenerative hyperplasia, which can cause NCPH, LSM did not differentiate between those patients with clinical features of portal hypertension and those without [6].

Guidelines recommend regular screening for varices by gastroscopy in cirrhotic patients with clinically significant portal hypertension, so that prophylactic therapy with nonselective beta blockers, or direct therapy with banding, can be given to prevent variceal haemorrhage [7]. There is little data on the prophylaxis and management of variceal bleeding in patients with NCPH. Largely by extrapolation from studies in cirrhosis and portal hypertension due to extrahepatic portal vein thrombosis, guidelines recommend the same approach to prophylaxis and treatment of bleeding as with patients with cirrhosis [8, 9].

Whilst gastroscopy is generally a safe procedure, it can be unpleasant for patients, there are risks related to conscious sedation, and it is relatively resource-intensive. In 2016, the Baveno consensus committee proposed that patients with cirrhosis who had LSM <20 kPa and platelet count >150 × 10^9^/L were at low risk of clinically significant varices and could defer gastroscopy, with annual monitoring of these parameters and referral for screening gastroscopy if the criteria were met [10]. These criteria had a low false negative rate (i.e., low risk of missing clinically significant varices) but when applied in practice, relatively few gastroscopies were avoided [11]. Expanding these criteria to include patients with LSM <25 kPa and platelet count >110 × 10^9^/L was shown to safely spare more unnecessary gastroscopies, at least amongst patients with cirrhosis due to hepatitis C, alcoholic, and nonalcoholic steatohepatitis [12]. However, the aetiology of liver disease may influence reliability of these noninvasive assessments, as subsequent studies in patients with different aetiologies of liver disease suggested an increased risk of missing clinically significant varices when extended Baveno criteria were applied [13, 14].

Although the Baveno and expanded Baveno have been the most prominent criteria proposed to stratify risk of varices, alternate criteria have been proposed by other groups. These include platelet count/spleen length ratio, which identified patients with portal hypertension due to cirrhosis or portal vein thrombosis at low risk of oesophageal varices, and LSM-spleen length/platelet count ratio scores [15, 16]. However, to date, stratification of risk of varices has been limited to patients with portal hypertension due to cirrhosis. The aim of this study was to evaluate whether these, or other criteria, may stratify risk of clinically significant varices in patients with NCPH.

## 2. Materials and Methods

This was a retrospective observational study. The study population comprised patients seen at a single tertiary referral centre between 2007 and 2018 for management of NCPH. To identify potential subjects for this study, a database search of electronic patient records was performed using key words “non-cirrhotic portal hypertension”, “NCPH”, “sarcoid”, “schistosomiasis”, “nodular regenerative hyperplasia”, “NRH”, “congenital hepatic fibrosis”, “portal vein thrombosis” and “Budd Chiari”. Inclusion criteria were clinical features of portal hypertension (splenomegaly, thrombocytopaenia, or endoscopic stigmata of portal hypertension); at least one clinic visit between 2007 and 2018; gastroscopy performed within 12 months of clinical assessment. Exclusion criteria were biopsy-proven cirrhosis; additional causes of liver disease; alcohol intake >20g/day for women; >30g/day for men; or malignancy involving the liver.

Subjects who met the inclusion/exclusion criteria were selected for further chart review. Information was collected on demographics; clinical events; LSM; laboratory results; pathology (fibrosis score); radiology (spleen size); endoscopy (gastroscopy date, presence and description of gastroesophageal varices). For the purpose of this study, varices were classed as high or low risk. Oesophageal varices that were described as small (< Grade 2) were classed as low risk varices (LRV). High risk varices (HRV) included oesophageal varices described as moderate/large size (≥ Grade 2); presence of red signs; varices banded by the endoscopist; or any gastric varices. Scores which indicate severity of liver disease (MELD, Childs Pugh) and fibrosis (FIB4, APRI) were calculated. Combinations of variables which have been shown to predict risk of varices in cirrhotic portal hypertension were also calculated, specifically platelet count/spleen length ratio (PSL) and liver stiffness-spleen length/platelet count ratio (LSP) [15, 16].

Prior to commencement of the study, the study protocol was reviewed and approved by the Institutional Research Ethics Board.

### 2.1. Statistical Analyses

Data were grouped according to presence or absence of HRV at gastroscopy. Normality was assessed using D'Agostino-Pearson test for Normal distributions. Normally distributed variables were expressed as mean ± standard deviation (sd), whilst non-normally distributed variables were expressed as median (interquartile range; IQR). For continuous variables, Student's t-test (for parametric data) or Mann-Whitney U test (for nonparametric data) were used to assess whether there were differences in demographics, fibrosis score, or severity of liver disease between patients with and without HRV. For noncontinuous variables, Chi squared or Fisher's exact tests were used. Variables which might associate with presence of HRV were analysed by univariate and multivariate analyses. Correlations were assessed by calculation of the Pearson correlation coefficient. To assess the performance of spleen size and PSL to predict presence of HRV at gastroscopy within the next 12 months, receiver operating characteristic (ROC) curves were constructed and analysed. Positive and negative predictive values were calculated using an estimated HRV prevalence of 30%. A *p* value of <0.05 was used to determine statistical significance. Statistical analyses were performed using MedCalc statistical software (version 17.9.7, Ostend, Belgium).

## 3. Results

### 3.1. Study Population

**Figure 1:**
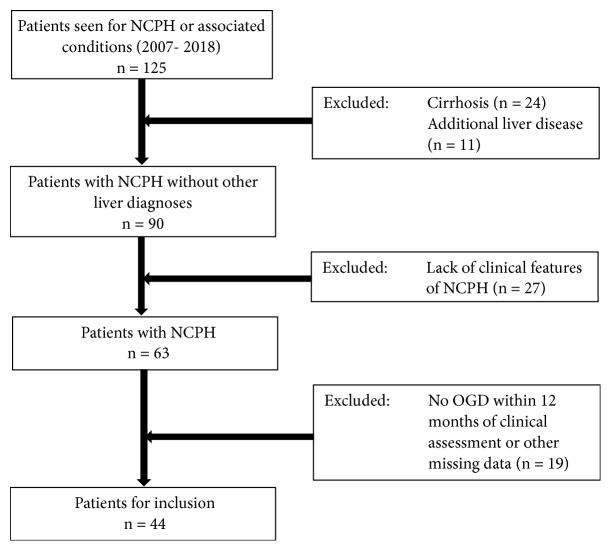
*Flow chart of patients evaluated for inclusion in the study. *NCPH, non-cirrhotic portal hypertension; OGD, oesophogastroduodenoscopy.

During the study period, 125 patients were identified who were seen at our institution for management of NCPH. Of these patients, 46 did not meet the inclusion criteria (27 due to lack of clinical features of portal hypertension; 19 without recorded gastroscopy within 12 months of assessment or other missing data) and 35 met exclusion criteria (cirrhosis in 24; additional liver disease in 11). In total 44 patients remained for inclusion in the study (Figure 1).

### 3.2. Patient Demographics

Of the 44 patients who met inclusion criteria, mean age was 46.8±16.7 years. Twenty-one were male and 23 were female. Median duration of follow-up was 83.7 months (IQR 40.9 – 123.0 months). In terms of ethnic background, 32 (73%) identified themselves as Caucasian, 4 (9%) as Black African, 3 (7%) as South Asian, 3 (7%) as Far East Asian, 1 (2%) as Arabic, and 1 (2%) as Latin American. Main causes of NCPH in this cohort were nodular regenerative hyperplasia (n = 16); congenital hepatic fibrosis (n = 8); idiopathic (n = 6); schistosomiasis (n = 6); and portal vein thrombosis (n = 5). Uncommon causes included myelofibrosis, cystic fibrosis, and sarcoid (1 case each). At gastroscopy, 15 patients (34%) had HRV, 15 patients (34%) had small varices, and 14 patients (32%) had no detectable varices. For the purposes of further analysis, patients with no varices and small varices were combined in the LRV group. There was a history of prior variceal bleeding in 30% of patients (13/44); 53% (8/15) of the HRV group; and 17% (5/29) of the LRV group.

There were no significant differences in patient demographics between the HRV and LRV groups (Table 1). Liver fibrosis (assessed by liver biopsy, where available, or as estimated by FIB4 or APRI scores) and severity of liver disease (NaMELD; Childs-Pugh Score) did not differ significantly between those with and without HRV (Table 1).

During the period of follow-up, three patients (7%) had an episode of variceal bleeding (all in the HRV group), four patients (9%) developed ascites (three in the HRV group, one in the LRV group), and four patients (9%) died (one in the HRV group, three in the LRV group). Causes of death were heart failure, septic shock, and renal failure. No patient died from variceal haemorrhage or associated complications.

### 3.3. Predictors of HRV in Patients with NCPH

We hypothesized that clinical parameters associated with portal hypertension may predict presence of HRV in patients with NCPH. The clinical parameters tested were spleen size, platelet count, LSM, and combinations of these variables which have previously been demonstrated to associate with HRV in patients with cirrhosis. These were platelet count-spleen length ratio (PSL) and liver stiffness-spleen length/platelet count ratio (LSP) [15, 16]. On univariate analysis, spleen size and PSL correlated with presence of varices but LSM, platelet count, and LSP did not (Table 2). In a multivariate model, PSL was an independent predictor of the presence of HRV only when spleen size was excluded, and vice versa. For spleen size, *β* = 0.35,* p *= 0.02; OR 1.42, 95% CI 1.06-1.92. For PSL, *β* = -1.47,* p *= 0.02; OR 0.23, 95% CI 0.07-0.80. As expected, a modest correlation between these two covariates was confirmed (r = -0.388;* p* = 0.013).

### 3.4. Performance of Spleen Size and PSL in Predicting Presence of HRV

**Figure 2:**
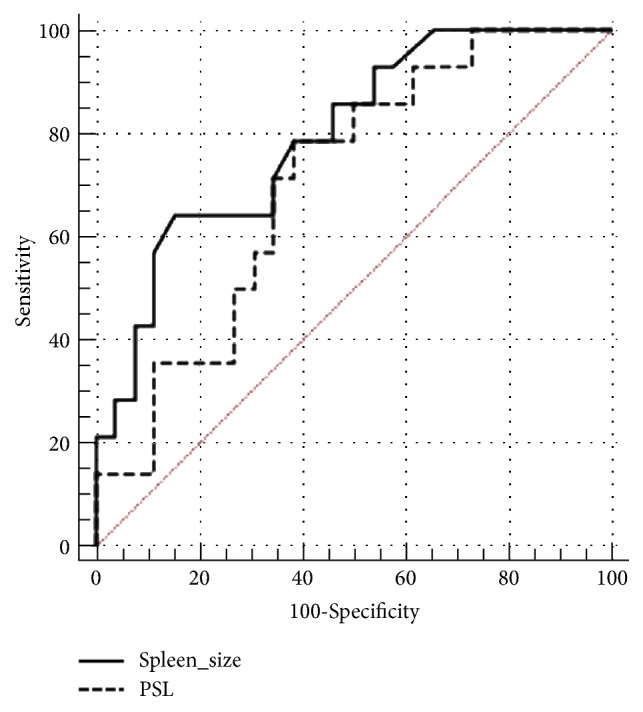
*Comparison of AUROC for spleen size and PSL (platelet count/spleen length ratio) in prediction of HRV at gastroscopy.* AUROC for spleen size was 0.809 (95% CI 0.658 – 0.913;* p* < 0.001). AUROC for PSL was 0.706 (95% CI 0.541 – 0.839,* p* = 0.014). The *p* value for the comparison was 0.400.

To assess the performance of spleen size and PSL in predicting presence of HRV at gastroscopy, ROC curves were constructed and analysed. Performances of these two parameters are summarised in Table 3. At a prevalence of HRV of 0.34, the AUROC of spleen size and PSL values for predicting presence of HRV were 0.809 (95% CI 0.658 – 0.913;* p* < 0.001) and 0.706 (95% CI 0.541 – 0.839,* p* = 0.014), respectively. Optimal thresholds, calculated to minimise the false negative rate, were spleen size >17.2 cm and PSL ≤ 5.21. Spleen size > 17.2 cm had a sensitivity of 78.57%, specificity 64.29%, PPV 48.53%, and NPV 87.50% for predicting the presence of HRV at gastroscopy within 12 months. PSL ≤ 5.21 had sensitivity 78.57%, specificity 61.54%, PPV 46.68%, and NPV 87.01% for presence of HRV at gastroscopy within 12 months. No statistically significant difference was found between AUROC for spleen size and PSL (Figure 2). Therefore, combining platelet count with spleen size in the PSL calculation did not add to the predictive value of spleen size alone with regard to risk of HRV within 12 months.

## 4. Discussion

To our knowledge, this is the first study to assess the role of noninvasive assessments in prediction of HRV in patients with NCPH. This study demonstrated that, in patients with NCPH, spleen size may predict presence of HRV at gastroscopy within 12 months. Adding platelet count (in the form of platelet count-spleen length ratio) did not add significantly to the predictive value of spleen size alone in this cohort. Notably, LSM, platelet count, and LSM-spleen length/platelet count ratio score, which may predict HRV in patients with cirrhosis [10, 15, 16], or simple fibrosis markers such as APRI and FIB-4, had no predictive role in our cohort of patients with NCPH.

Our finding that LSM is not associated with HRV in patients with NCPH is in line with findings of another small series of patients with nodular regenerative hyperplasia, which reported no association between LSM and gastroesophageal varices [6]. The number of patients with HRV in this cohort however was very small. LSM has been proposed as an adjunctive tool to distinguish between cirrhosis and idiopathic NCPH as the values are generally higher than healthy controls but lower than matched patients with cirrhosis [17, 18]. Complicating the issue is the diverse range of causes of NCPH. For example, infiltrative liver diseases and congenital hepatic fibrosis might be expected to be associated with high LSM. Others, such as nodular regenerative hyperplasia and idiopathic NCPH, are associated with less liver stiffness and elevations in LSM may be more reflective of increased portal flow, although chronic portal hypertension may result in secondary liver fibrosis [6]. Our results support previous observations by others that LSM is not associated with HVPG in patients with NCPH [17] and confirm that LSM is not associated with HRV in a group of patients with diverse causes of NCPH.

The prevalence of HRV in our study is lower than in some other reported series [17, 18]. This may reflect the heterogeneity of causes of NCPH in our patient cohort, as different causes of NCPH may be associated with varying risk of varices or variceal bleeding [6, 19]. This study included both patients newly diagnosed with NCPH and patients under follow-up, including 30% that had previously been treated for gastroesophageal varices, whilst other published series have tended to report rates of gastroesophageal varices at first diagnosis. Whilst variceal haemorrhage is a common and significant complication of NCPH, bleeding is by no means universal at diagnosis (for example, in one large series, 43% of patients had no history of bleeding at diagnosis of NCPH [20]). We believe that our patient cohort, comprising a combination of new and follow-up patients with NCPH, is relevant to a real-world clinical setting where risk of HRV may change over time and requires ongoing clinical evaluation.

Morbidity and mortality associated with variceal bleeding are less in NCPH than in patients with cirrhosis, likely due to preserved liver function and avoidance of complications related to hepatic decompensation [9]. However, risks associated with variceal bleeding are still such that evaluation of potential predictive tests should focus on minimising of false negatives, so that patients at high risk of variceal bleeding are not falsely classified in the low risk group. Selecting a cut-off value to minimise false negatives, our results found that spleen size >17.2 cm is associated with 87.5% NPV for HRV at gastroscopy within 12 months. Applying this cut-off to our cohort, 18 patients (62%) of patients with LRV could have avoided unnecessary screening gastroscopy, but 3 patients (20%) with HRV would have been missed. Recently, addition of spleen stiffness ≤46 kPa to the Baveno VI criteria (LSM < 20 kPa and platelet count >150 × 10^9^/L) was found to increase the proportion of screening gastroscopies which could be avoided in patients with cirrhosis, whilst minimising missed HRV (<5%) [21]. Whether spleen stiffness may be useful in prediction of HRV in patients with NCPH, alone or in combination with other clinical parameters, remains to be evaluated.

This study has some limitations. The retrospective nature of the study carries inherent limitations of bias, which may be reflected in the relatively high number of patients excluded due to lack of a gastroscopy within 12 months of clinical assessment. This may have been due to a clinical impression of mild disease based on other clinical parameters. Few episodes of variceal bleeding occurred during follow-up, so we are unable to explore associations between clinical variables and risk of variceal haemorrhage. However gastroesophageal varices classified as high risk in this study (variceal size ≥ Grade 2; presence of red signs; gastric varices of any size) are associated with increased risk of variceal haemorrhage [10]. This definition of HRV has been widely used by others as a surrogate endpoint in other similar studies (for example, [21–23]). Categorisation of varices into high and low risk groups is in line with distinctions used in clinical practice to define gastroesophageal varies which are clinically significant and require therapy [10]. Use of high versus low risk varices as an outcome variable is subject to subjective assessment and inter-observer variation. However, this is a real-world study and the approaches employed reflect routine clinical practice and decision-making in management of patients with NCPH, usually based on these endoscopic criteria.

## 5. Conclusions

In patients with NCPH, spleen size may predict risk of HRV at gastroscopy within 12 months. Unlike in patients with portal hypertension related to cirrhosis, LSM and platelet count are not useful to assess risk of HRV in patients with NCPH. Recently, spleen stiffness has been incorporated into assessments of variceal risk in patients with cirrhotic hypertension. Spleen stiffness, alone or in combination with other clinical parameters such as spleen size, warrants evaluation in patients with NCPH to further refine stratification of variceal risk.

## Figures and Tables

**Table 1:** Demographics of study participants. HRV, high risk varices; LRV; low risk varices; NRH, nodular regenerative hyperplasia; CHF, congenital hepatic fibrosis; PVT, portal vein thrombosis; OGD, oesophogastroduodenoscopy; MELD, Model for End-stage Liver Disease score; APRI, AST to platelet ratio index; LSM, liver stiffness measurement. Unless otherwise stated, values are median (IQR).

**Patient demographics **	**All patients **	**With HRV **	**Without HRV **	**P value **
	**(n = 44) **	**(n = 15) **	**(n = 29) **	
**Age** (years; mean ± s.d.)	46.7 ± 16.7	52.1 ± 15.1	45.5 ± 17.2	0.219

**Gender **				
Male (%)	21 (48)	8 (53)	12 (41)	0.532
Female (%)	23 (52)	7 (47)	17 (59)	

**BMI**	23.0 (21.0 – 26.0)	23.0 (19.8 – 27.0)	23.0 (21.0 – 26.0)	0.854

Aetiology				
NRH (%)	16 (36)	6 (40)	10 (34)	-
CHF (%)	8 (18)	0 (0)	8 (28)	-
Schistosomiasis (%)	6 (14)	4 (27)	2 (7)	-
Idiopathic (%)	6 (14)	2 (13)	4 (14)	-
PVT (%)	5 (11)	2 (13)	3 (10)	-
Other (%)	3 (7)	1 (7)	2 (7)	-
Time to OGD (days)	83.5 (30 – 121)	63 (36 - 121)	94 (29 - 124)	0.815

**Liver disease Severity**				
MELD	9.0 (8.0 – 12.0)	9.0 (8.0 – 11.0)	9.0 (8.0 – 12.0)	0.661
Childs Pugh A (%)	38 (86)	13 (87)	25 (86)	0.966
Childs Pugh B (%)	6 (14)	2 (13)	4 (14)	

**Fibrosis**				
Laennec score at liver biopsy (mean ± s.d.)	1.19 ± 1.05	1.18 ± 1.08	1.16 ± 1.07	0.953
FIB4	3.21 (1.66 – 5.01)	3.17 (1.66 – 5.41)	3.07 (1.55 – 4.68)	0.541
APRI	0.97 (0.53 – 1.55)	0.89 (0.54 – 1.78)	0.97 (0.51 – 1.53)	0.789
LSM (kPa)	8.7 (6.7 – 11.5)	8.9 (4.8 – 10.3)	8.6 (7.1 – 11.9)	0.438

**Table 2:** *Association of liver stiffness and clinical parameters of portal hypertension with presence of HRV at gastroscopy.* HRV, high risk varices; LRV, low risk varices; LSM, liver stiffness measurement; PSL, platelet count to spleen length; LSP, liver stiffness-spleen length to platelet count ratio score. Values are median (IQR), unless stated otherwise.

Variable	All patients (n = 44)	HRV (n = 15)	LRV (n = 29)	P value
LSM (kPa)	8.7 (6.7 – 11.5)	8.9 (4.8 – 10.3)	8.6 (7.1 – 11.9)	0.438

Platelet count (x10^9^)	90 (60 – 128)	76 (56 – 99)	110 (62 – 132)	0.182

Spleen size (cm; mean ± s.d.)	17.54 ± 4.10	20.48 ± 4.19	16.07 ± 3.22	0.001

PSL	5.24 (3.94 – 7.92)	4.35 (2.77 – 5.21)	6.42 (4.23 – 9.86)	0.033

LSP	1.41 (1.10 – 2.16)	1.78 (1.41 – 2.11)	1.27 (0.96 – 3.15)	0.300

**Table 3:** *Performances of spleen size and PSL in predicting HRV at gastroscopy within 12 months in patients with NCPH.* Sensitivity, specificity, PPV, and NPV values are calculated for a threshold of >17.2cm for spleen size and ≤ 5.21 for PSL, with HRV prevalence of 30%. AUROC, area under receiver-operator characteristic curve; PPV, positive predictive value; NPV, negative predictive value; PSL, platelet count-spleen length ratio.

Variable	AUROC (95% CI)	Sensitivity (%)	Specificity (%)	PPV (%)	NPV (%)
Spleen size	0.809 (0.658 – 0.913)	78.57	64.29	48.53	87.50

PSL	0.706 (0.541 – 0.839)	78.57	61.54	46.68	87.01

## Data Availability

The data used to support the findings of this study are available from the corresponding author upon request.
